# Neuraminidase-1 contributes significantly to the degradation of neuronal B-series gangliosides but not to the bypass of the catabolic block in Tay–Sachs mouse models

**DOI:** 10.1016/j.ymgmr.2015.07.004

**Published:** 2015-08-15

**Authors:** Z.K. Timur, S. Akyildiz Demir, C. Marsching, R. Sandhoff, V. Seyrantepe

**Affiliations:** a Izmir Institute of Technology, Department of Molecular Biology and Genetics, Izmir, Turkey; b Lipid Biochemistry Lab, Cancer Research Center, Heidelberg, Germany

**Keywords:** Tay–Sachs, Hexosaminidase, Ganglioside, Neuraminidase

## Abstract

Tay–Sachs disease is a severe lysosomal storage disorder caused by mutations in the HEXA gene coding for α subunit of lysosomal β-Hexosaminidase A enzyme, which converts G_M2_ to G_M3_ ganglioside. HexA^−/−^ mice, depleted of the β-Hexosaminidase A iso-enzyme, remain asymptomatic up to 1 year of age because of a metabolic bypass by neuraminidase(s). These enzymes remove a sialic acid residue converting G_M2_ to G_A2,_ which is further degraded by the still intact β-Hexosaminidase B iso-enzyme into lactosylceramide. A previously identified ganglioside metabolizing neuraminidase, Neu4, is abundantly expressed in the mouse brain and has activity against gangliosides like G_M2_*in vitro*. Neu4^−/−^ mice showed increased G_D1a_ and decreased G_M1_ ganglioside in the brain suggesting the importance of the Neu4 in ganglioside catabolism. Mice with targeted disruption of both HexA and Neu4 genes showed accumulating G_M2_ ganglioside and epileptic seizures with 40% penetrance, indicating that the neuraminidase Neu4 is a modulatory gene, but may not be the only neuraminidase contributing to the metabolic bypass in HexA^−/−^ mice. Therefore, we elucidated the biological role of neuraminidase-1 in ganglioside degradation in mouse. Analysis of HexA^−/−^ Neu1^−/−^ and HexA^−/−^ Neu4^−/−^ Neu1^−/−^ mice models showed significant contribution of neuraminidase-1 on B-series ganglioside degradation in the brain. Therefore, we speculate that other neuraminidase/neuraminidases such as Neu2 and/or Neu3 might be also involved in the ganglioside degradation pathway in HexA^−/−^ mice.

## 1 Introduction

Tay–Sachs disease is the second common lysosomal storage disorder caused by mutations in the HEXA gene [1]. The HEXA gene encodes the α-subunit of the lysosomal β-Hexosaminidase A (HexA) [E.C3.2.1.52] enzyme which removes N-acetyl-galactosamine residues from the G_M2_ ganglioside converting it to the G_M3_ ganglioside for further degradation. Tay–Sachs patients suffer from progressive neuronal degeneration, muscle weakness, blindness and epilepsy and affected with the severe infantile form, they die in the second to the fourth year of their life [2]. The β-Hexosaminidase A deficient mouse model of Tay–Sachs disease (HexA^−/−^) does not mimic the human disease because the ganglioside degradation pathway differs between mice and humans [3], [4], [5], [6], [7]. The behaviors and the motor coordination of these mice were the same as the wild type mice (HexA^+/+^) until at least 1 year of life span. Only a limited accumulation of G_M2_ ganglioside and the membranous cytoplasmic bodies were observed in the brain of HexA^−/−^ mice. This inequality revealed that there may be a bypass in the G_M2_ ganglioside degradation. Instead of going through the G_M3_ pathway, G_M2_ ganglioside may be degraded *via* the glycolipid G_A2_ (asialo-form of G_M2_ ganglioside), which bypasses the G_M3_ dependent degradation [3]. It has been proposed that the activities of neuraminidase(s) [E.C.3.2.1.18], which remove the sialic acid residue from the G_M2_ ganglioside converting it to the G_A2_ ganglioside that is further degraded by β-Hexosaminidase B enzyme to lactosylceramide, take part in the bypassing pathway of the G_M2_ ganglioside degradation [5], [7]. A previously identified ganglioside metabolizing neuraminidase-4 is abundantly expressed in the mouse brain and has activity against gangliosides like G_M2_, *in vitro*. In the mouse model with the neuraminidase Neu4 deficiency, lysosomal storage was shown in the lung and the spleen with microscopic investigation. In addition, abnormal ganglioside patterns (increased GD_1a_ and decreased G_M1_ levels) in the brain of mice were shown by thin layer chromatography analysis [8]. To elucidate whether neuraminidase Neu4 is involved in the G_M2_ ganglioside degradation *in vivo*, a mouse model with combined deficiencies of β-Hexosaminidase A and neuraminidase-4 (HexA^−/−^ Neu4^−/−^) was generated. Double knockout mice have multiple degenerating neurons in the cortex and hippocampus and multiple layers of cortical neurons accumulate G_M2_ ganglioside. The significantly higher level of the G_M2_ ganglioside in the lysosomes of neurons was shown in HexA^−/−^ Neu4^−/−^ mice. More importantly mice with the HexA and Neu4 enzyme deficiency had epileptic seizures (the hallmark of Tay–Sachs patients), which were not observed in mice with a single HexA enzyme deficiency. Since only 40% of all mice with the double enzyme deficiency had seizures, we postulated that the Neu4 is a modulatory gene and neuraminidase Neu4 is not the only neuraminidase contributing to the metabolic bypass seen in the HexA deficient mice [9]. Therefore, we suggested that another neuraminidase and/or neuraminidases such as lysosomal neuraminidase Neu1 might have a role in metabolic bypass. In this study we generated and analyzed the mice models with deficiencies of three enzymes; β-Hexosaminidase A, neuraminidase-1 and neuraminidase-4, to investigate the contribution of neuraminidase(s) on the ganglioside degradation in mice.

## 2 Material and methods

### 2.1 Animals

Triple mice with three enzyme deficiencies (HexA^−/−^ Neu4^−/−^ Neu1^−/−^) were previously generated by Prof. Dr. Volkan Seyrantepe in Montreal, Canada and donated by Prof. Alexey V. Pshezhetsky (Centre Hospitaliere Universitaire Sainte-Justine, University of Montreal, Montreal, Quebec, Canada). These mice were generated by breeding double knockout mice with the deficiency of β-Hexosaminidase A and neuraminidase-4 (HexA^−/−^ Neu4^−/−^) with hypomorphic mouse model (CathA^*S190A-Neo*^ mice was named as Neu1^−/−^ mice in this study) with reduced neuraminidase-1 activity [10]. Later on, triple mice were breed with wild type mice strain C57/Black6 to further obtain single (Neu4^−/−^, HexA^−/−^, Neu1^−/−^) and double deficient (HexA^−/−^ Neu4^−/−^ and HexA^−/−^ Neu1^−/−^) mice as well as triple deficient mice in the same genetic background. All mice were bred and maintained in the Turkish Council on Animal Care (TCAC) accredited animal facility of Izmir Institute of Technology according to the TCAC guidelines. Mice were housed under constant temperature and humidity on a 12 h light:dark cycle. The animal care and the use in the experiments were granted by the Animal Care and Use Committee of Ege University, Izmir, Turkey.

### 2.2 Genotyping

All mice were genotyped by PCR for three alleles from the genomic DNA extracted from mice's tail. The PCR for Neu4 and HexA alleles [9] and Neu1 [10] was performed as previously described.

### 2.3 Lipid isolation and ganglioside purification

Age matched single, double and triple deficient mice as well as control mice (3, 6 and 9 months old) were sacrificed by cervical dislocation. 100 mg brain tissue from their right and left cerebral hemispheres was removed, immediately frozen on dry ice and kept at − 80 °C until needed. To isolate total lipids and purify ganglioside from brain tissue an optimized form of Folch lipid extraction method was used as previously described [9], [11].

### 2.4 Thin layer chromatography and orcinol staining

Isolated gangliosides were run according to a previous method [9]. Orcinol (Sigma-447420) dye was dissolved with 25% sulfuric acid in glass TLC sprayer (Sigma). The dye was sprayed on the plates and incubated at 120 °C for 10 min. The gangliosides were identified by comparing them with the brain ganglioside standards (Avanti Polar Lipids). Images of plates were taken with the VersaDoc™ Imaging System for quantification.

### 2.5 Mass spectrometric analysis of gangliosides

For mass spectrometric analysis, lipids were extracted by a different procedure. 3 ml of methanol was added to 300 mg brain tissue and homogenized in a glass vial with a Heidolph Silent Crusher M Politron Homogenizer. 3 ml of chloroform was added to the suspension and the mixture was incubated at 37 °C in an ultra sound bath by turning on the ultra sound 3 times for 3 min within the 15 min incubation period. Samples were centrifuged at 2000 rpm at RT and the supernatant was taken in a new glass vial. Extraction of the tissue pellet was repeated once with 2 ml chloroform:methanol:water (10:10:1) and once with chloroform:methanol:water (30:60:8). Supernatants were combined and evaporated with N_2_. Lipid samples were used by Prof. Roger Sandhoff's laboratory (German Cancer Research Center) for mass spectrometric analysis.

### 2.6 Mass spectrometric analysis of lipids

Lipids were extracted from samples as mentioned above. Synthetic lipid standards were purchased from Avanti Polar Lipids. Chemicals and solvents were purchased from Sigma-Aldrich. All standards and lipid extracts were dissolved in an appropriate volume of 5 mM ammonium formate in methanol–chloroform 5:1 (v/v) just prior to analysis. Mass spectrometric analyses were conducted on a hybrid triple quadrupole/linear ion trap 4000 QTRAP instrument (Applied Biosystems/MDS Sciex, Ontario, Canada) which is equipped with an electrospray ionization source. Lipid solutions were infused into the electrospray source at a 15 μl/min flow rate. The instrument was used either in the single-stage MS mode or in the tandem MS mode (product ion or precursor ion). The spray was operated for the detection of positive mode ions 264.4 for ceramide species [12] and 184.4 for phosphatidylcholine and sphingomyelin species [13]. The Precursor scan studies were operated using the following 4000 QTRAP instrumental parameters: curtain gas (CUR), 10.00 arbitrary units (arb); ion spray voltage (IS), 3500.00; temperature (TEM), 0.00 °C; ion source gas 1 (GS1), 10.00; ion source gas 2 (GS2), 0.00; collision gas (CAD), 6.00 arb; collision energy (CE), 52 eV; Collision Cell Exit Potential (Cxp), 9; Declustering potential (DP), 60 V; Entrance potential, 10. For each spectrum were collected as a sum of the 20 multichannel analysis (MCA) scans during 3.350 min time period.

### 2.7 Immunohistochemical analysis

For immunohistochemical analysis, mice were anesthetized and transcardiac perfusion was initiated with phosphate-buffered saline (PBS) followed by 4% paraformaldehyde in PBS. Brain tissues were removed and placed in the same fixative overnight at 4 °C, and then treated sequentially with 10%, 20% and 30% sucrose in PBS overnight at 4 °C. Brains were embedded in OCT (Sakura) and kept at − 80 °C until used. Ten micrometer sections were taken by Leica Cryostat (CM1850-UV) at − 20 °C and the G_M2_ ganglioside was immunostained as previously described by KM966 primer and DyLight 488 secondary antibody (Thermo) [9]. Slides were studied on fluorescent Microscopy (Olympus BX53).

### 2.8 Expression analysis

Expression analysis of neuraminidases (Neu1, Neu2, Neu3 and Neu4) [14] and HexB in 3 and 6 month old HexA^−/−^, Neu4^−/−^, HexA^−/−^ Neu4^−/−^, HexA^−/−^ Neu4^−/−^ Neu1^−/−^ as well as wild type mice was done with the Roche LightCycler 480 system using Roche LightCycler 480 SYBR Green I Master Mix after RNA was extracted from 100 mg brain tissue by TRIzol Reagent (Invitrogen) and then cDNA was synthesized by NEB Protoscript M-MuLvTaq RT PCR kit. GAPDH gene was used as internal control, 3 mice were analyzed from each group and all samples were run in duplicate. 40 ng of cDNA was used in the 20 μl reaction mix containing 20 pmol of each primer and 1 × Roche LightCycler 480 SYBR Green I Master Mix. Conditions for PCR were; 1 cycle 10 min at 95 °C; 45 cycles 20 s at 95 °C, 15 s at 61 °C, 22 s at 72 °C, reading was done after each cycle. In the end 1 cycle 30 s at 95 °C, 10 s at 60 °C then continuous reading was applied while temperature increases to 99 °C to detect primer dimer if exists. The following pairs of primers were used for expression analysis; Neu1F: 5′-TCATCGCCATGAGGAGGTCCA, Neu1R:5′-AAAGG GAATGCCGCTCACTCCA, Neu2F: 5′-CGCAGAGTTGATTGTCCTGA, Neu2R: 5′-TTCTGA GCAGGGTGCAGTTTCC, Neu3F: 5′-CTCAGTCAGAGATGAGGATGCT, Neu3R: 5′-GTGAGACATA GTAGGCATAGGC, Neu4F: 5′-AGGAGAACGGTGCTCTTCCAGA, Neu4R: 5′-GTTCTTGCCAG TGGCGATTTGC, HexBF: 5′-AGTGCGAGTCCTTCCCTAGT, HexBR: 5′-ATCCGGACATCGTTTGGTGT, GADPHF: 5′-CCCCTCATTGACCTCAACTAC, GADPHR: 5′-ATGCATTGCTGACAATCTTGAG.

### 2.9 Enzyme assays

Neuraminidase (Sigma 69587), β-glucosidase (SIGMA M3633), β-galactosidase (SIGMA M1633) and α-L-iduronidase (SC220961) enzyme activities in the brain were assayed by using the corresponding fluorogenic 4-methylumbelliferyl substrates as previously described [15]. 50 mg brain tissue from 6 month old WT, HexA^−/−^, Neu4^−/−^, HexA^−/−^ Neu4^−/−^ and HexA^−/−^ Neu4^−/−^ Neu1^−/−^ mice was homogenized in 0.4 M sodium acetate (pH 4.3) buffer by a mini homogenizer and sonicated at 60 V for 10 s. 10 μl of homogenate was incubated in a sodium acetate buffer that has 0.5 mM substrate; reaction was stopped with 0.2 M glycine buffer (pH 10.8) after 0.5 h of incubation at 37 °C. Samples were read *via* a spectrofluorometer at excitation wavelength of 365 nm and emission wavelength of 445 nm. Protein concentration in the sample was measured by Bradford reagent (Sigma) and specific enzyme activity was calculated.

## 3 Results

### 3.1 Generation of single, double and triple deficient mice

Previously generated HexA^−/−^ Neu4^−/−^ Neu1^−/−^ male mice were crossed with C57/Black6 female mice. Mice were genotyped for 3 different genes (HexA, Neu4 and Neu1) by PCR method as previously described [9], [16]. Mice with desired genotypes were crossed with each other to get single (HexA^−/−^, Neu4^−/−^, and Neu1^−/−^), double (HexA^−/−^ Neu4^−/−^ and HexA^−/−^ Neu1^−/−^) and triple deficient (HexA^−/−^ Neu4^−/−^ Neu1^−/−^) mice in the same genetic background. Mated mice heterozygotes for 3 genes gave offspring at the expected Mendelian ratio (1:2:1) and generated triple deficient mice were healthy and lived longer than 2 years. There were no significant differences in weight gain between mutant and littermate control mice. Both males and female triple deficient mice were fertile.

### 3.2 Altered ganglioside levels in both HexA^−/−^ Neu1^−/−^ and HexA^−/−^ Neu4^−/−^ Neu1^−/−^ mice

**Fig. 1:**
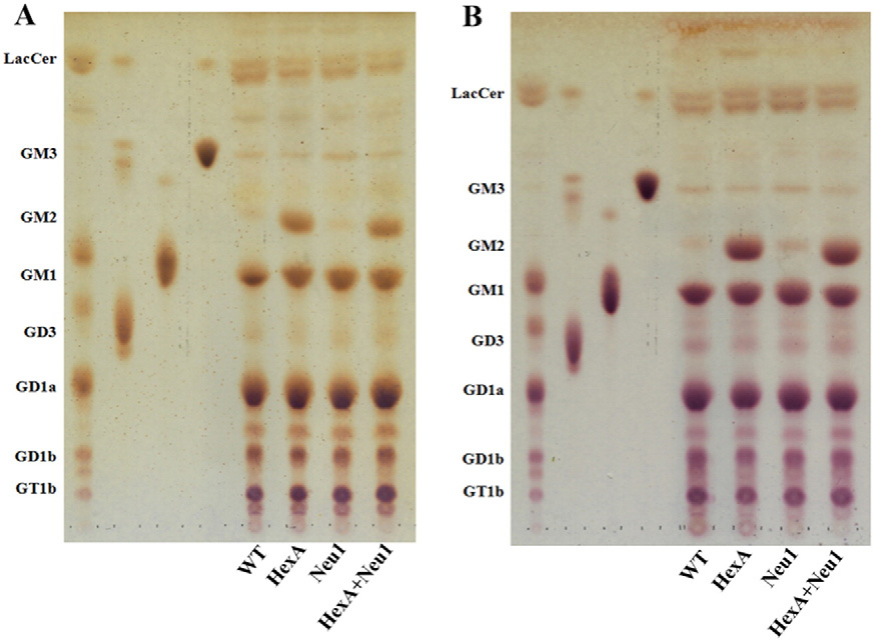
Thin layer chromatography and orcinol staining for gangliosides of 3 month (A) and 6 month (B) HexA + Neu1 double deficient mice with its wild type and single HexA and Neu1 deficient counterparts.

Thin layer chromatography analysis of the brain gangliosides showed that 3 month old single HexA^−/−^ and double HexA^−/−^ Neu1^−/−^ mice had an increased level of G_M2_ ganglioside in comparison to wild type mice (Fig. 1). In HexA^−/−^ mice, the accumulation of the G_M2_ ganglioside was previously shown [3], [4], [5]. Although we determined a slightly increased level of G_D1_ ganglioside (~ 1.3 fold) in HexA^−/−^ Neu1^−/−^ mice in comparison to HexA^−/−^ mice, the levels of G_M1_ and G_M2_ in the gangliosides were not different in these mice's brain. To compare the effect of age on accumulation, 6 month old mice's brain gangliosides were also analyzed. We found that the G_M2_ ganglioside level in the 6 month old mice's brain was not significantly different than that of the 3 month old mice (Fig. 1). However, in the detailed analysis of 6 month old mice's brain gangliosides with mass spectrometry, we showed that HexA^−/−^ Neu1^−/−^ mice have a significant increased level of G_D2_ ganglioside (~ 3 fold) and G_D1_ ganglioside (~ 1.3 fold), and a relatively decreased level of G_D3_ ganglioside (~ 0.8 fold), in comparison to HexA^−/−^ mice whereas G_M1_, G_M2_, G_M3_, and G_T1_ gangliosides and SM4 sulfatide levels remained similar to HexA^−/−^ mice (Fig. 4A). Besides the similarities in the levels of G_M1_ and G_M2_ gangliosides in both mice, there was a relative decrease in the ratio of G_M2_/G_M1_ gangliosides in HexA^−/−^ Neu1^−/−^ mice than that of HexA^−/−^ mice (Fig. 4B).

**Fig. 2:**
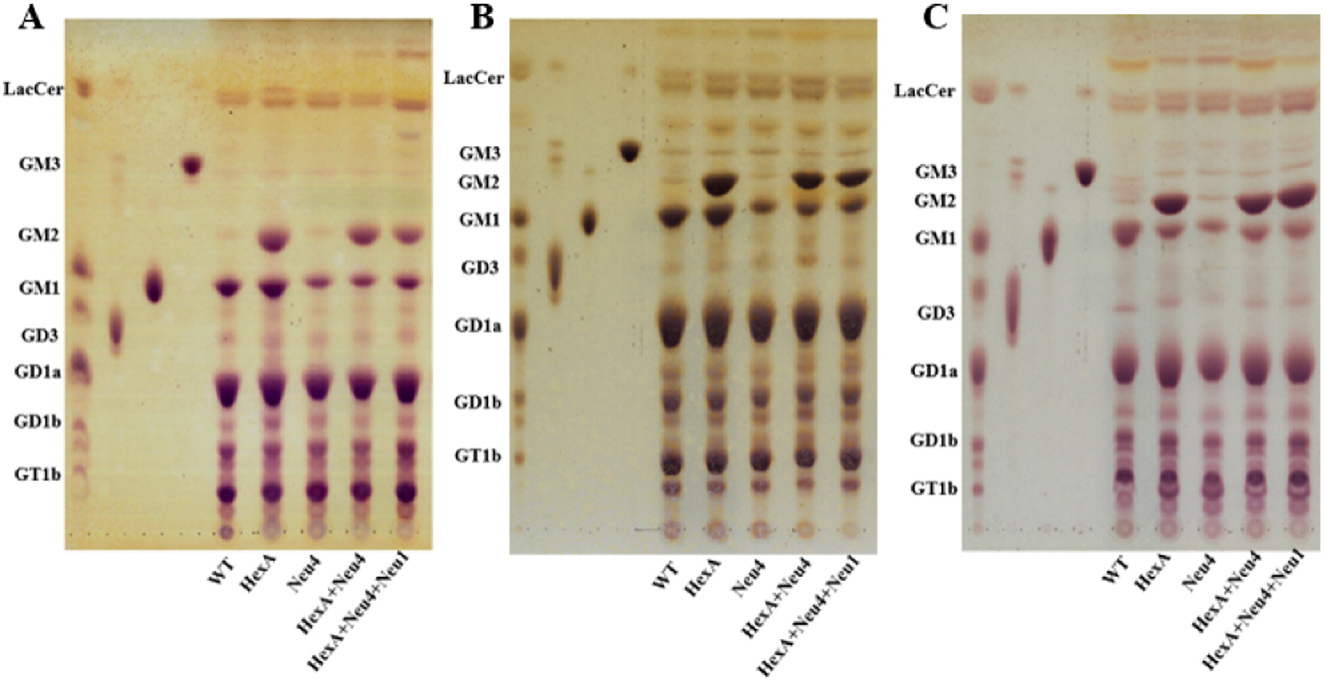
Thin layer chromatography and orcinol staining for gangliosides of 3 month (A), 6 month (B) and 9 month (C) old triple deficient (HexA + Neu4 + Neu1) mice with its wild type, single HexA and Neu4 deficient and double HexA + Neu4 deficient counterpart.

**Fig. 3:**
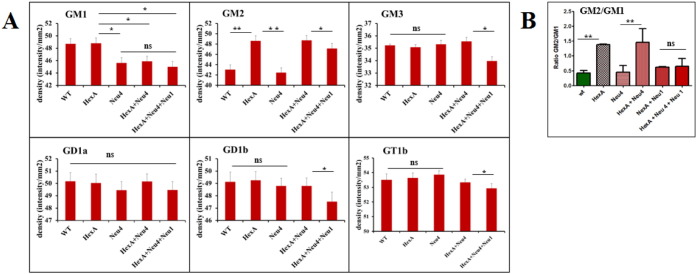
(A) Relative G_M1_, G_M2_, G_M3_, G_D1a_, G_D1b_, and G_T1b_ levels and (B) GM2/GM1 ratio of 6 month old HexA + Neu4 + Neu1 mice with wild type, single HexA and Neu4 deficient, double HexA + Neu4 and HexA + Neu1 deficient mice brain analyzed with thin layer chromatography (n = 3, *p < 0.05, **p < 0,01).

**Fig. 4:**
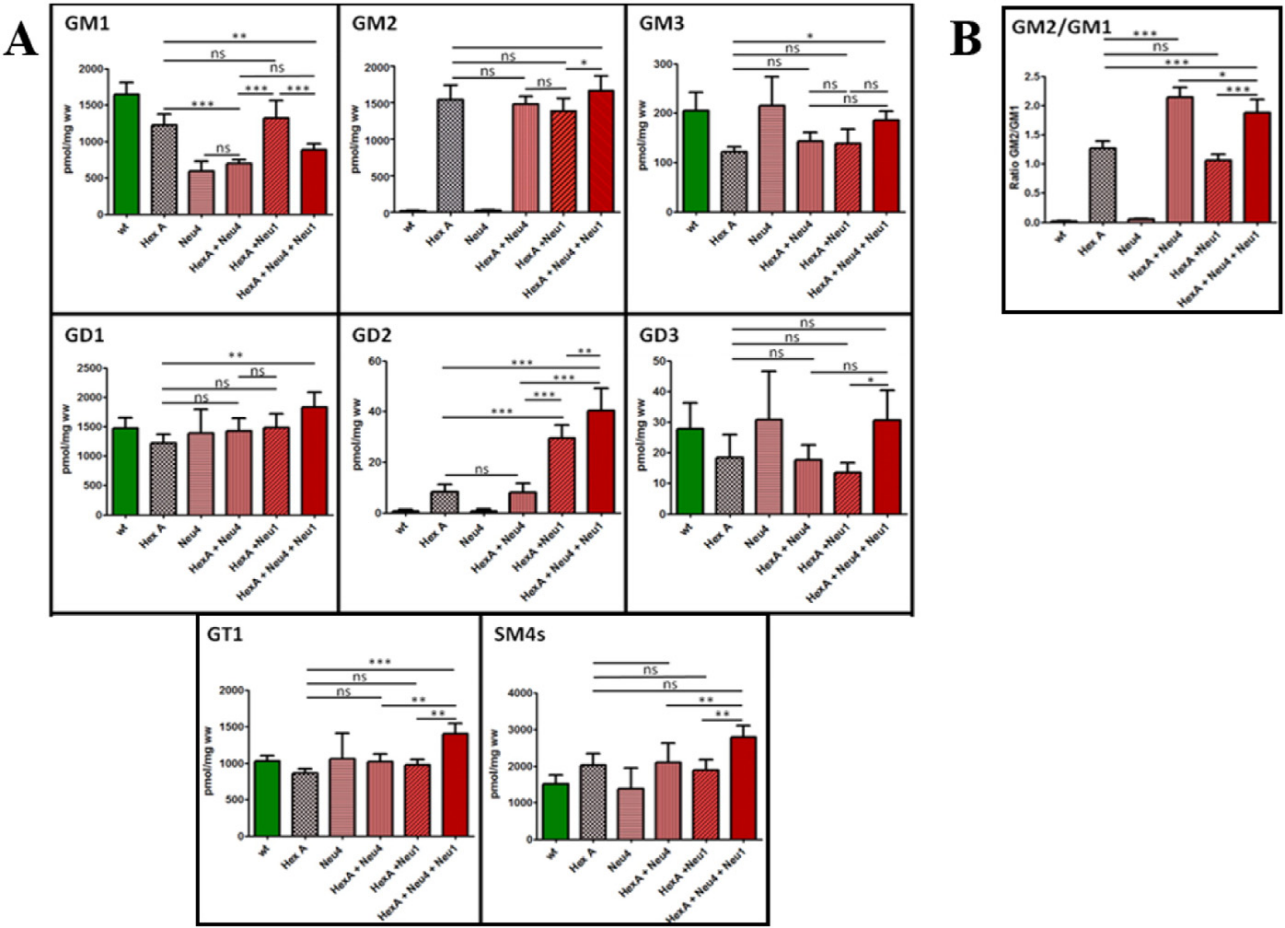
(A) Relative G_M1_, G_M2_, G_M3_, G_D1_, G_D2_, G_D3_, G_T1_ and SM4 levels and (B) GM2/GM1 ratio of 6 month old HexA + Neu4 + Neu1 mice with wild type, single HexA and Neu4 deficient, double HexA + Neu4 and HexA + Neu1 deficient mice brain analyzed with mass spectrometry (n = 3; ns = not significant; *p < 0.05; **p < 0.01; ***p < 0.001).

Neuraminidase-4 deficiency in three different mice (Neu4^−/−^, HexA^−/−^ Neu4^−/−^ or HexA^−/−^ Neu4^−/−^ Neu1^−/−^) caused a decreased level of G_M1_ ganglioside that could be determined by both thin layer chromatography (Fig. 2) and mass spectrometric (Fig. 4) analysis, in comparison to wild type and age-matched HexA^−/−^ mice as previously shown due to the lack of the activity of the neuraminidase-4 on ganglioside G_D1a_ [8]. Although HexA^−/−^ Neu4^−/−^ mice showed ~ 2 fold increase in the level of the G_M2_ ganglioside compared to wild type and HexA^−/−^ mice in all 3, 6 and 9 month old age groups, as previously shown [9], HexA^−/−^ Neu4^−/−^ Neu1^−/−^ mice showed very similar G_M2_ ganglioside level in comparison to HexA^−/−^ Neu4^−/−^ mice both in thin layer chromatography (Fig. 2 and Fig. 3) and mass spectrometric analysis (Fig. 4). Accumulation level of the G_M2_ ganglioside is indicated by the ratio of G_M2_/G_M1_ ganglioside. While in the HexA^−/−^ mice the G_M2_/G_M1_ ratio is almost ~ 1.3 fold, and this ratio increases above ~ 2 fold in HexA^−/−^ Neu4^−/−^ mice. We determined that HexA^−/−^ Neu4^−/−^ Neu1^−/−^ mice had a higher G_M2_/G_M1_ ratio than the HexA^−/−^ mice but lower ratio than HexA^−/−^ Neu4^−/−^ mice which could be related to the slightly increased level of G_M1_ ganglioside by the deficiency of the neuraminidase-1 (Fig. 4A and B).

Although the levels of G_M2_ ganglioside in brain were similar in 3 month and 6 month old HexA^−/−^ Neu4^−/−^ and HexA^−/−^ Neu4^−/−^ Neu1^−/−^ mice, mass spectrometry analysis revealed that HexA^−/−^ Neu4^−/−^ Neu1^−/−^ mice had an altered ganglioside profile in general content. The HexA^−/−^ Neu4^−/−^ Neu1^−/−^ mice with lack of β-Hexosaminidase and neuraminidase-4 and reduced neuraminidase-1 activity (10% activity) showed significant increased level of G_M3_ ganglioside (~ 1.5 fold), G_D1_ ganglioside (~ 1.2 fold), G_D2_ ganglioside (~ 4 fold), G_T1_ ganglioside (~ 1.6 fold), G_D3_ ganglioside (~ 1.2 fold) and the glycolipid sulfatide (SM4s) (~ 1.2 fold) than that of HexA^−/−^ mice (Fig. 4). These findings on the ganglioside level changes indicate the possible role of neuraminidase-1 and neuraminidase-4 on the ganglioside degradation pathway in mice and also emphasize the importance of neuraminidase-1 activity especially on b-series ganglioside catabolism since reduced activity results in higher amount of G_D1_, G_D2_, G_D3_ and G_T1_ gangliosides.

### 3.3 Altered secondary lipid composition in both HexA^−/−^ Neu4^−/−^ and HexA^−/−^ Neu4^−/−^ Neu1^−/−^ mice

**Fig. 5:**
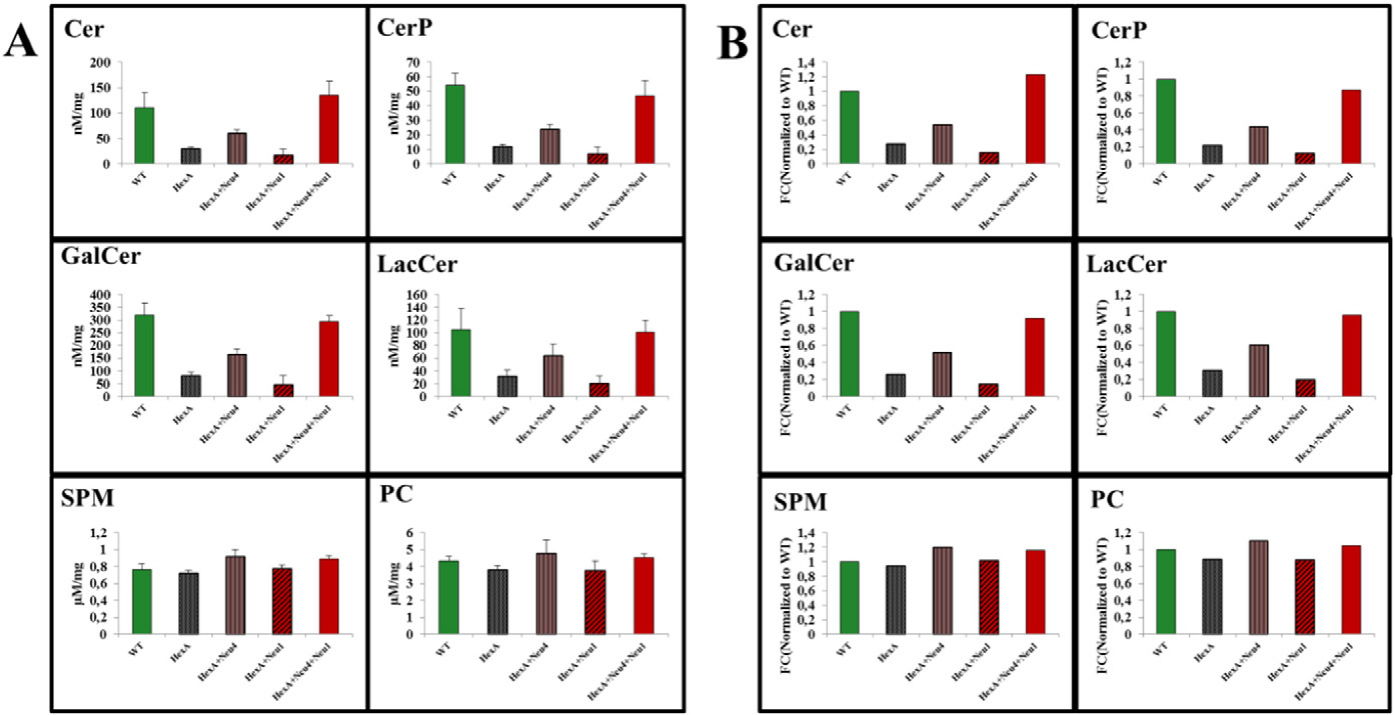
(A) Relative and (B) fold changes of ceramide (Cer), ceramide phosphate (CerP), galactosylceramide (GalCer), lactosylceramide (LacCer), sphingomyelin (SPM) and phosphatidylcholine (PC) levels of 9 month old HexA + Neu4 + Neu1 mice with wild type, single HexA deficient, double HexA + Neu4 and HexA + Neu1 deficient mice brain analyzed with mass spectrometry (n = 3; p < 0.005).

Effects of β-Hexosaminidase and neuraminidase(s) deficiency on the secondary lipid composition in brain tissue were also studied. Whole brain tissue from 9 month old Wt, HexA^−/−^, HexA^−/−^ Neu1^−/−^, HexA^−/−^ Neu4^−/−^, and HexA^−/−^ Neu4^−/−^ Neu1^−/−^ mice and their control groups was analyzed with mass spectrometry. Phosphatidylcholine (PC) and sphingomyelin (SPM) species were monitored by parent ion scanning of m/z 184.4 in positive ion mode. Ceramide (Cer), and ceramide species such as ceramide phosphate (CerP), galactosylceramide (GalCer) and lactosylceramide (LacCer) were analyzed by parent ion scanning of m/z 264.4 in positive ion mode. Both β-Hexosaminidase A and neuraminidase-4 deficiency caused a decrease in level of CerP, GalCer and LacCer in brain tissue compared to WT mice. We also determined ~ 1.06 fold increase in the ceramide level in the Neu4^−/−^ mice (data not shown). All of the ceramide species levels were slightly lower in both HexA^−/−^ Neu4^−/−^ and HexA^−/−^ Neu4^−/−^ Neu1^−/−^ mice compared to WT mice, whereas HexA^−/−^ Neu4^−/−^ Neu1^−/−^ deficient mice had elevated amount of ceramide content compared to HexA^−/−^ Neu4^−/−^ mice and Wt mice which indicates the possible role of neuraminidase-1 in the ganglioside degradation pathway (Fig. 5A).

Mass spectrometry analysis of SPM and PC species in the 9 month old mice brains revealed that HexA^−/−^ Neu4^−/−^ mice had ~ 1.2 and ~ 1.11 fold increase in SPM and PC content compared to WT mice. Similarly there was ~ 1.16 and ~ 1.05 fold increase in SPM and PC amount in HexA^−/−^ Neu4^−/−^ Neu1^−/−^ mice brain compared to WT mice brain. These results may also indicate the possible role of neuraminidase-1 in the SPM and PC metabolism. Deficiency of neuraminidase-1 in addition to β-Hexosaminidase A and neuraminidase-4 leads to an increase in the SPM but a decrease in the PC level in the mouse brain (Fig. 5B).

### 3.4 Increased G_M2_ ganglioside accumulation in the brain cortex of HexA^−/−^, HexA^−/−^ Neu4^−/−^, HexA^−/−^ Neu4^−/−^ Neu1^−/−^ mice

**Fig. 6:**
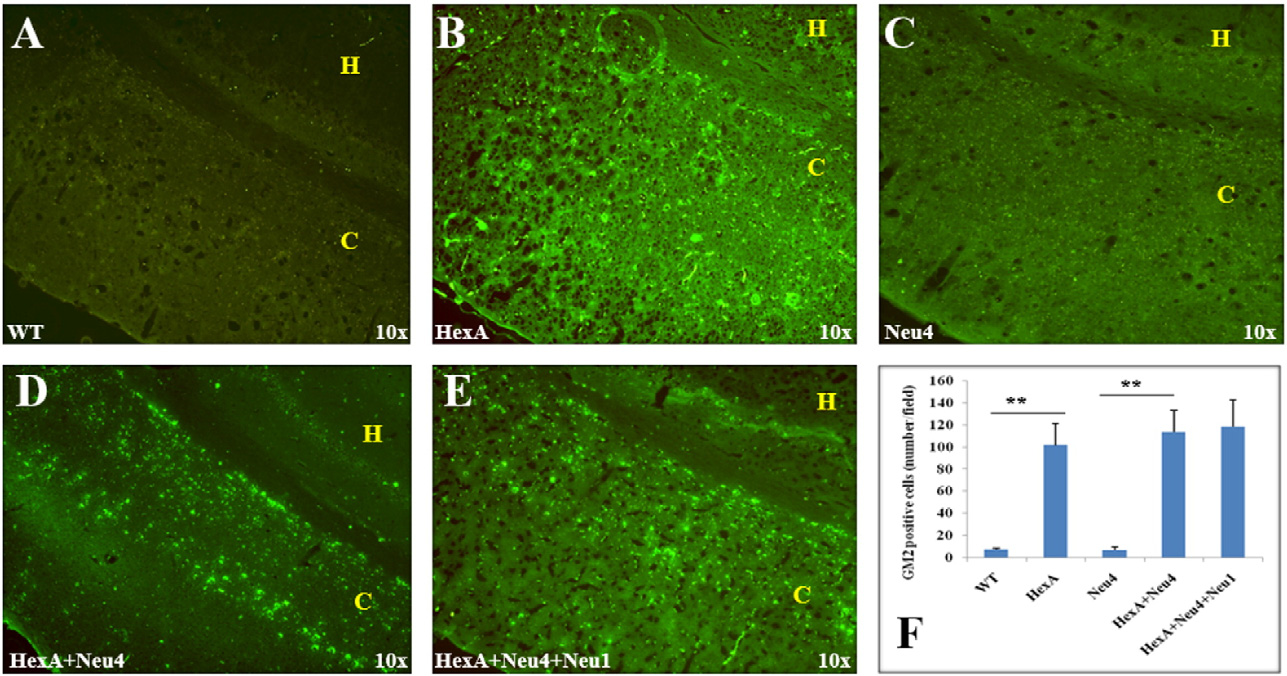
Accumulation of GM2 ganglioside in 3 month old HexA (B) HexA + Neu4 (D) and HexA + Neu4 + Neu1 (E) deficient mice brain. Wild type (A) and Neu4 deficient (C) do not show GM2 accumulation neither in cortex (indicated by yellow C) nor in hippocampus (yellow H) region of brain. (F) Semiquantitative analysis of GM2 positive cells by US NIH image software program (ImageJ) (n = 3, **p < 0.01).

**Fig. 7:**
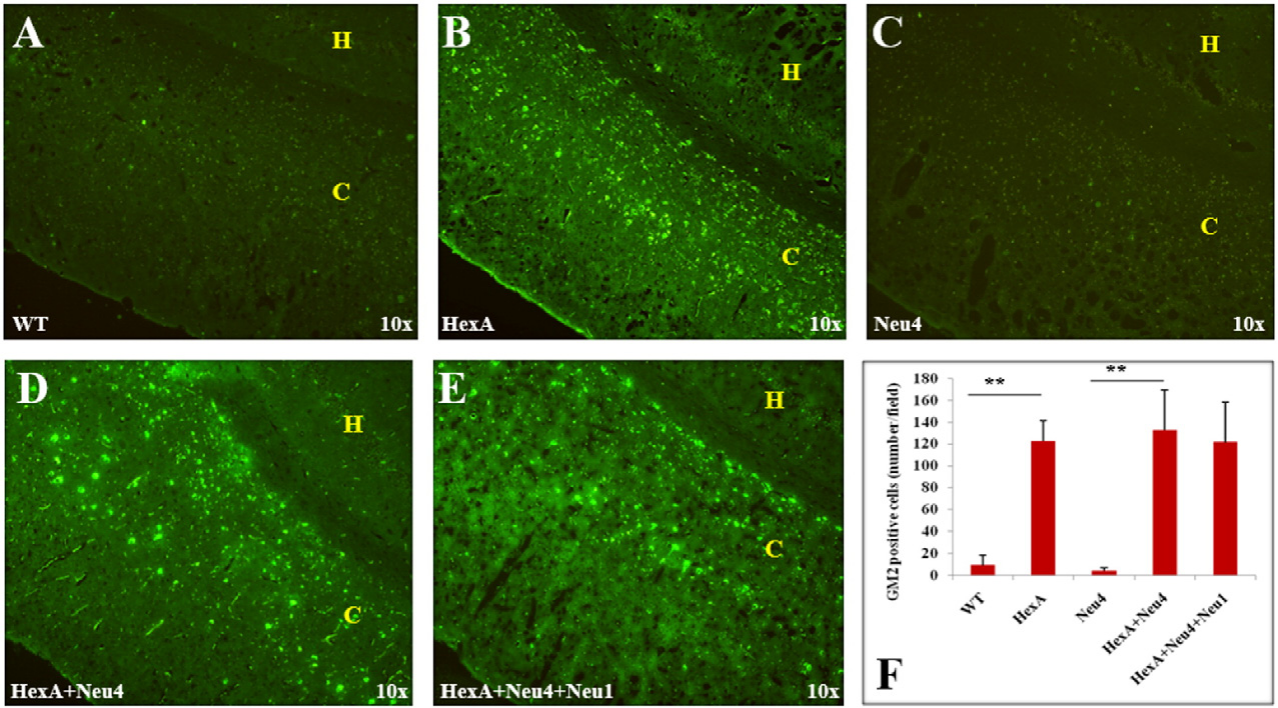
Accumulation of GM2 ganglioside in 6 month old HexA (B) HexA + Neu4 (D) and HexA + Neu4 + Neu1 (E) deficient mice brain. Wild type (A) and Neu4 deficient (C) do not show GM2 accumulation neither in cortex (indicated by yellow C) nor in hippocampus (yellow H) region of brain. (F) Semiquantitative analysis of GM2 positive cells by US NIH image software program (ImageJ) (n = 3, **p < 0.01).

**Fig. 8:**
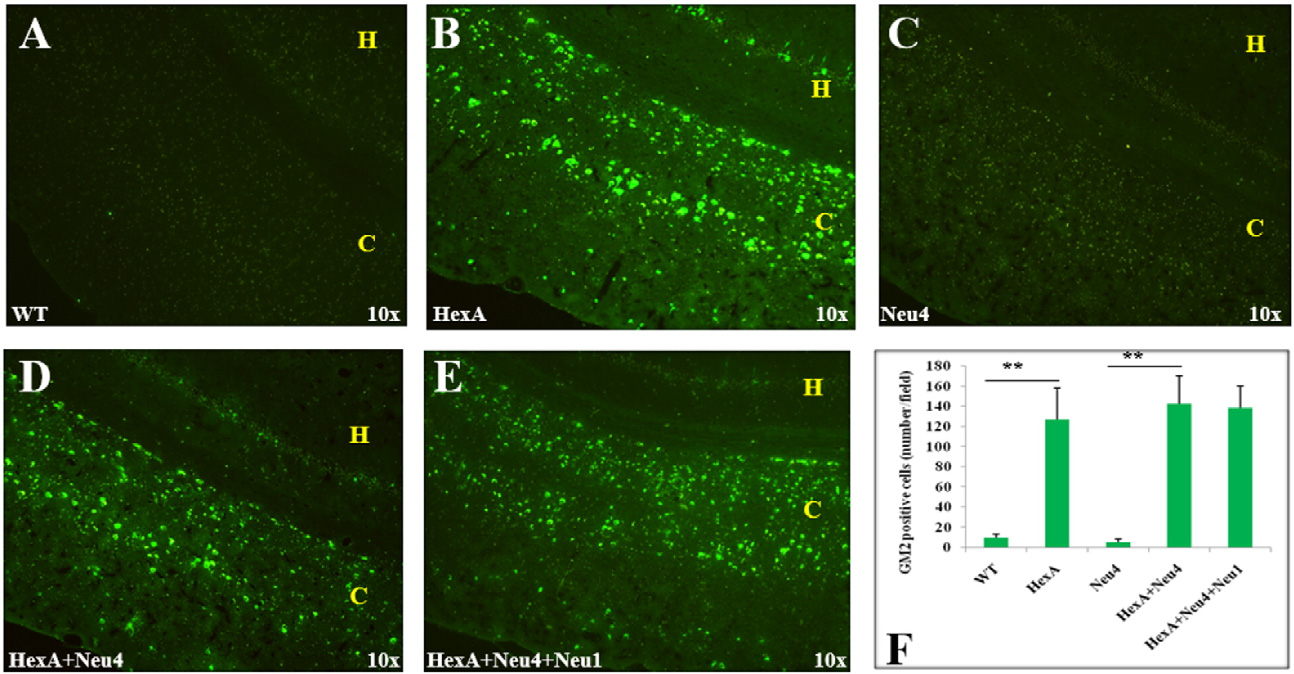
Accumulation of GM2 ganglioside in 9 month old HexA (B) HexA + Neu4 (D) and HexA + Neu4 + Neu1 (E) deficient mice brain. Wild type (A) and Neu4 deficient (C) do not show GM2 accumulation neither in cortex (indicated by yellow C) nor in hippocampus (yellow H) region of brain. (F) Semiquantitative analysis of GM2 positive cells by US NIH image software program (ImageJ) (n = 3, **p < 0.01).

**Fig. 9:**
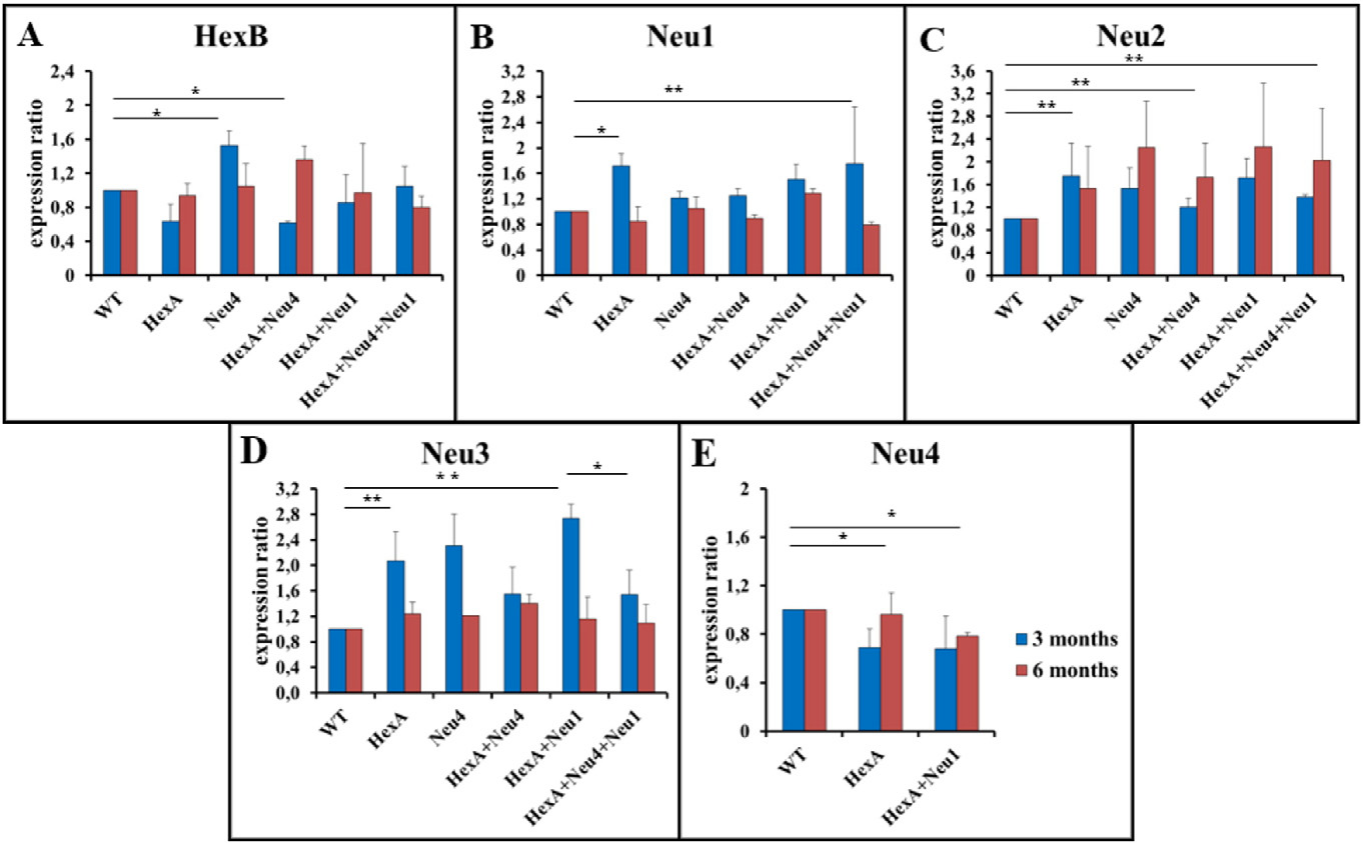
Relative expressions of HexB (A) and Neu1 (B), Neu2 (C), Neu3 (D), Neu4 (E) sialidases in 3 and 6 month old HexA + Neu4 + Neu1 mice with wild type, single HexA and Neu4 deficient and double HexA + Neu4 deficient mice brains (n = 3, *p < 0.05, **p < 0,01).

Accumulation of the G_M2_ ganglioside in the coronal sections of the brain was also studied by immunohistochemistry analysis using the human–mouse chimeric monoclonal antibody, KM966 [9]. No G_M2_ ganglioside accumulation was detected in the brain of wild type or Neu4^−/−^ mice from 3 months (Fig. 6), 6 months (Fig. 7) and 9 months (Fig. 8). However in the hippocampus of HexA^−/−^, HexA^−/−^ Neu4^−/−^ and HexA^−/−^ Neu4^−/−^ Neu1^−/−^ mice, relatively high level of G_M2_ accumulation was detected compared to the cortex. G_M2_ ganglioside specific immunostaining of the brain revealed that although HexA^−/−^ Neu4^−/−^ mice and HexA^−/−^ Neu4^−/−^ Neu1^−/−^ mice had higher amount of accumulated G_M2_ ganglioside than HexA^−/−^ mice, the regions for accumulation were similar in the brains of different deficient mice.

### 3.5 Altered neuraminidase(s) expression levels in HexA^−/−^, HexA^−/−^ Neu4^−/−^, HexA^−/−^ Neu4^−/−^ Neu1^−/−^ mice

Expression analysis of four mammalian neuraminidases (Neu1, Neu2, Neu3 and Neu4) and HexB enzyme, isomer of HexA, in the brain of the deficient mice revealed that deficiency of any neuraminidase in the mouse brain triggers the expression of other neuraminidase(s). HexB expression level decreased when the HexA gene is defective in the 3 month old mice brain, but significantly increased in the 3 month old Neu4^−/−^ mice and 6 month old HexA^−/−^ Neu4^−/−^ mice (Fig. 9A). The neuraminidase-1 expression in the 3 month old (HexA^−/−^, Neu4^−/−^, HexA^−/−^ Neu4^−/−^, HexA^−/−^ Neu1^−/−^ and HexA^−/−^ Neu4^−/−^ Neu1^−/−^) mice were higher than control, whereas slightly increased expression level was detected in 6 month old mice HexA^−/−^ Neu4^−/−^ Neu1^−/−^ (Fig. 9B). In HexA^−/−^ Neu4^−/−^ Neu1^−/−^ mice the disrupted gene was not the neuraminidase-1 gene, instead the protective protein Cathepsin A (PPCA) coding gene expression was interrupted by inserting neomycin cassette inserted in its non-coding region. Neomycin insertion caused a decrease in the PPCA mRNA level consistent with the reported hypomorphic (partial loss of function) effects of the neomycin gene. Neuraminidase-1 makes a complex with β-galactosidase and PPCA in lysosomes, therefore the decrease of the PPCA gene expression does not cause changes in expression level of neuraminidase-1 directly but results in high reduction of neuraminidase-1 activity [17].

Expression of neuraminidase-2 is increased in both 3 and 6 month old mice in all genotypes. Expression level was significantly higher especially in 6 month old HexA^−/−^ Neu4^−/−^ Neu1^−/−^ mice as compared to that of 3 month old mice (Fig. 9C). Neuraminidase-3 expression showed higher level in 3 month old HexA^−/−^, Neu4^−/−^ and HexA^−/−^ Neu1^−/−^ mice as compared to HexA^−/−^ Neu4^−/−^ and HexA^−/−^ Neu4^−/−^ Neu1^−/−^ mice (Fig. 9D). No significant difference in level of neuraminidase-3 expression between 3 month old HexA^−/−^ Neu4^−/−^ and HexA^−/−^ Neu4^−/−^ Neu1^−/−^ mice was detected however in the 6 month old mice there was a lower level of neuraminidase-3 expression in HexA^−/−^ Neu4^−/−^ Neu1^−/−^ mice. Neuraminidase-4 expression was only studied in HexA^−/−^ and HexA^−/−^ Neu1^−/−^ since Neu4 gene is deleted in Neu4^−/−^, HexA^−/−^ Neu4^−/−^ and HexA^−/−^ Neu4^−/−^ Neu1^−/−^ mice. Lower expression of neuraminidase-4 was detected in both age groups in HexA^−/−^ and HexA^−/−^ Neu1^−/−^ mice brain (Fig. 9E).

### 3.6 Altered specific activity of neuraminidase-1, β-glucosidase, β-galactosidase and α-L-iduronidase in deficient mice

**Fig. 10:**
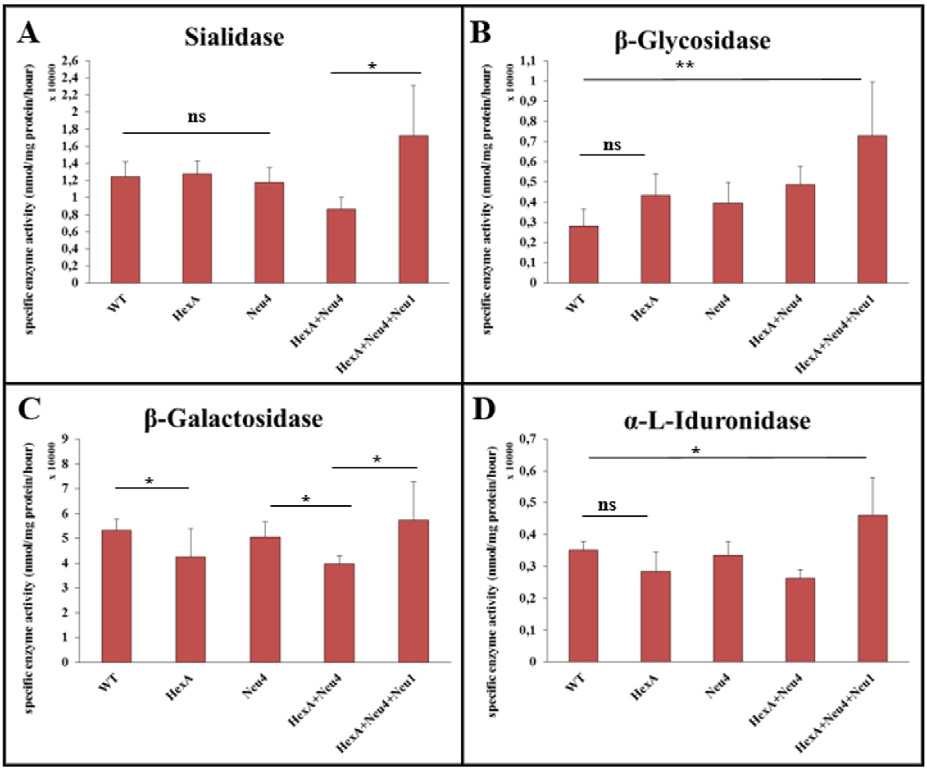
Specific enzyme activities of sialidase (A), β-glycosidase (B), β-galactosidase (C), α-L-iduronidase (D) in 6 month old HexA + Neu4 + Neu1 mice with wild type, single HexA and Neu4 deficient and double HexA + Neu4 deficient mice brains (n = 3, ns = not significant, *p < 0.05, **p < 0,01).

Neuraminidase activity against artificial substrate 4-MU-N-acetilneuraminic acid was measured to determine whether the increased level of neuraminidase-2 and neuraminidase-3 expression results in an increase in the specific activity at protein level. No differences were detected for neuraminidase activity in HexA^−/−^ and Neu4^−/−^ mice compared to WT. However, 1.5 fold increased neuraminidase activity was detected in HexA^−/−^ Neu4^−/−^ Neu1^−/−^ mice (Fig. 10A). This increase might be related to the increased expression level of neuraminidase 2 and neuraminidase-3. In order to determine whether other independent lysosomal degradation pathways were affected from deficiencies of HexA, neuraminidase-4 or neuraminidase-1 enzymes; β-glucosidase, β-galactosidase and α-L-iduronidase enzyme activities were also measured. β-Glycosidase enzyme showed an increased activity especially in HexA^−/−^ Neu4^−/−^ Neu1^−/−^ mice (Fig. 10B). In HexA^−/−^ Neu4^−/−^ Neu1^−/−^ mice although there is an increase in the activity of β-galactosidase enzyme compared to HexA^−/−^ Neu4^−/−^, the activity is not significantly different from WT (Fig. 10C). α-L-iduronidase enzyme activity was also increased significantly in the HexA^−/−^ Neu4^−/−^ Neu1^−/−^ mice compared to WT and remained unchanged in other mice (Fig. 10D). Relatively low changes in the activities of these enzymes might be the result of secondary accumulated substrates of each enzyme in lysosomes.

## 4 Discussion

Tay–Sachs disease is the second most common lysosomal storage disorder in which G_M2_ ganglioside accumulates in the nerve cells due to the deficiency of lysosomal β-Hexosaminidase A (HexA) enzyme. Accumulation leads epileptic crisis, blindness, dementia, paralysis and even death in the early ages. It has been reported that the disruption of the HEX A gene in mouse embryonic stem cells resulted in a mice model that have no neurologic abnormalities up to one year of age [5]. The phenotypic differences between Tay–Sachs mouse model and Tay–Sachs patients suggested that the ganglioside degradation pathways in humans and mice are different. It was hypothesized that mouse neurons are enriched in a lysosomal ganglioside neuraminidase activity which removes the terminal sialic acid from G_M2_ ganglioside converting it into glycolipid G_A2_ which is further degraded by enzyme β-Hexosaminidase B [7]. The involvement of neuraminidase-4 in metabolic bypass was previously studied in mouse showing some (with epileptic crisis) but not all HexA^−/−^ Neu4^−/−^ mice have the increased accumulation of G_M2_ ganglioside in the brain cortex. Since 40% of HexA^−/−^ Neu4^−/−^ mice showed Tay–Sachs related features, we suggested that neuraminidase-4 is not only neuraminidase in metabolic bypass but may be other neuraminidase(s) also have role in this pathway along with neuraminidase-4 [9]. In the current work, we assessed whether lysosomal neuraminidase-1, with neuraminidase-4, is responsible *in vivo* for the metabolic bypass in the HexA deficient mouse model of Tay–Sachs disease by studying a mice model with the deficiencies of β-hexosaminidase A, neuraminidase-4 and reduced neuraminidase-1 (10% of normal activity).

Neuraminidase Neu1 had a strong G_M3_ and G_D1a_ ganglioside hydrolyzing activity, but weak activity toward G_M2_ ganglioside, *in vitro*. Besides, the impaired metabolism of G_M3_ ganglioside in cultured skin fibroblasts from sialidosis and galactosialidosis patients was reported with a storage of G_M3_ and G_D3_ gangliosides in visceral tissues but not in brain of sialidosis patients [17]. Ganglioside profiling with both thin layer chromatography and mass spectrometry analysis revealed that HexA^−/−^ Neu4^−/−^ Neu1^−/−^ mice had an altered ganglioside pattern, different from HexA^−/−^ Neu4^−/−^ mice, compatible with the previous studies that show neuraminidase-1 function on gangliosides. Although HexA^−/−^ Neu4^−/−^ mice had an increased G_M2_ and a decreased G_M1_ ganglioside level, HexA^−/−^ Neu4^−/−^ Neu1^−/−^ mice had increased level of G_M3_, G_D1_, G_D2_, G_D3_ and G_T1_ gangliosides with SM4 sulfatides which are the members of b series gangliosides [18]. Since the deficiency of neuraminidase-1 resulted in increased level of these b-series gangliosides, but not G_M2_ ganglioside, we suggest that neuraminidase-1 contributes the degradation of b-series gangliosides but not the metabolic bypass in HexA^−/−^ mice. Since HexA^−/−^ Neu1^−/−^ and HexA^−/−^ Neu4^−/−^ Neu1^−/−^ mice have 10% of normal activity neuraminidase-1, we speculate that the level of enzyme activity might be enough to degrade G_M2_ ganglioside in mice causing no excessive accumulation. To reveal the exact effect of neuraminidase-1 on ganglioside degradation *in vivo*, a previously generated knockout mouse model of neuraminidase Neu1 might be used to obtain HexA^−/−^ Neu1^−/−^ mice [16].

Studies of brain tissue from Tay–Sachs disease showed that one third of the dry weight to be ganglioside, mostly G_M2_ but there are also detectable amounts of asialo-G_M2_, LacCer, and glucocerebrosides [19]. In our study we showed that Cer, CerP, GalCer and LacCer amounts are higher in HexA^−/−^ Neu4^−/−^ as well as HexA^−/−^ Neu4^−/−^ Neu1^−/−^ mice brain compared to HexA^−/−^ mice although it was relatively lower than that of WT mice. These results showed that deficiency of neuraminidase-1 and neuraminidase-4 causes the accumulation of Cer, CerP, LacCer and GalCer compared to HexA^−/−^ mice. Myelin covers and facilitates the propagation of an electrical impulse down the axon to transmit information from one neuron to another. In many lysosomal storage disorder the myelin sheath deformation was reported [20]. GalCer, sulfatide, and sphingomyelin are structural components of myelin sheaths and major lipids in oligodendrocytes and Schwann cells in the brain [21]. Additionally, analysis of Tay–Sachs disease patient samples revealed accumulation of not only GM2 ganglioside but also secondary lipids for example phospholipids, cerebrosides, sphingomyelin, cholesterol and cholesterol esters, and so forth [19]. In our study, the accumulation of phosphatidylcholine and sphingomyelin in HexA^−/−^ Neu4^−/−^ and HexA^−/−^ Neu4^−/−^ Neu1^−/−^ was shown for the first time by mass spectrometry. The accumulation of phosphatidylcholine and sphingomyelin, was higher in the brain from HexA^−/−^ Neu4^−/−^ than HexA^−/−^ Neu4^−/−^ Neu1^−/−^ which indicates the possible role of neuraminidase-1 as well as neuraminidase-4 in the phosphatidylcholine and sphingomyelin metabolism.

Real-time PCR analysis for neuraminidases showed that neuraminidase-2 and neuraminidase-3 had an increased gene expression in all deficient mice (Fig. 9). The increased expression level of neuraminidase-2 and neuraminidase-3 in HexA^−/−^ Neu4^−/−^ mice was found as previously shown [9], while expression level in HexA^−/−^ Neu4^−/−^ Neu1^−/−^ mice was shown for the first time in this study. Although neuraminidase-3 is the most active neuraminidase on gangliosides [8] there was not a significant difference in neuraminidase enzyme activity in both HexA^−/−^ Neu4^−/−^ and HexA^−/−^ Neu4^−/−^ Neu1^−/−^ mice (Fig. 10). Also there is an increase in the expression level of neuraminidase-1 which may result from the accumulated substrates in the lysosome because of insufficient activity of neuraminidase-1. The cell may need more neuraminidase-1 activity and express neuraminidase-1 more than normal conditions but since there is not enough PPCA protein in the lysosome neuraminidase-1 cannot form an active complex and cannot degrade all substrates. When we combine the gene expression results with neuraminidase enzyme activity results, we speculate that this unchanged neuraminidase activity in HexA^−/−^ Neu4^−/−^ and HexA^−/−^ Neu4^−/−^ Neu1^−/−^ mice may occur from the increased gene expression of neuraminidase-2 and neuraminidase-3 in these two mice models. So in the absence of neuraminidase-4 and neuraminidase-1, neuraminidase-2 and/or neuraminidase-3 might have an increased function in the ganglioside degradation pathway (especially for G_M2_ ganglioside degradation that cannot completely degrade as a result of HexA enzyme deficiency) which causes G_M2_ ganglioside not to accumulate further in the HexA^−/−^ Neu4^−/−^ Neu1^−/−^ mice. Besides neuraminidase enzyme activity, we observed non-significant changes in other studied enzymes; β-glucosidase, β-galactosidase and α-L-iduronidase enzymes. We didn't expect to see increased activity of β-galactosidase enzyme in HexA^−/−^ Neu4^−/−^ Neu1^−/−^ mice since PPCA gene is defected but some studies showed that β-galactosidase has its own activity independently from the lysosomal multienzyme complex [22]. So we speculate that the low expression level of PPCA seems not to cause a complete loss of β-galactosidase enzyme activity in these mice models. The changes in enzymes' activity might be the result of altered lysosomal conditions because all degradation reactions take part in the lysosome. When high levels of macromolecules accumulate in lysosomes such as gangliosides, they inhibit other catabolic enzymes that are not genetically deficient due to secondary substrate accumulation [23]. These enzymes are responsible for the degradation of glucocerebroside, GM1 ganglioside and keratan sulfate and glycosaminoglycans respectively, so the increased activity of each enzyme might be the result of undegraded substrates that might be accumulated by the secondary effect of neuraminidase-1 deficiency. Detailed analysis for glucocerebroside, keratan sulfate and glycosaminoglycan accumulation in HexA^−/−^ Neu1^−/−^ and HexA^−/−^ Neu4^−/−^ Neu1^−/−^ mice helps us to better understand the function of neuraminidase-1 on substrate degradation with or without neuraminidase-4.

In this study we showed that although both neuraminidase-1 and neuraminidase-4 have function on ganglioside degradation *in vivo*, they differ in their ganglioside specificity. It was previously shown that in addition to lysosomal catabolism, neuraminidase-1 regulates various important cellular events through desialylation of surface molecules, like activating the phagocytosis in macrophages and dendritic cells [17], activating macrophages and forming a link to the cellular immune response [24], regulating insulin signaling [25] and regulating lysosomal exocytosis [26]. It was shown in many studies that gangliosides in the cell membrane function in cell to cell recognition, adhesion and especially in signal transduction [12], [21]. For instance, G_D1_ and G_T1_ gangliosides have functions in receptor tyrosine kinase activation, G_D2_ ganglioside involves in the activation of c-Met through MEK/Erk and PI3K/Akt signaling pathways and enhances cell migration and proliferation, G_M3_ ganglioside interacts with EGFR, FGFR and VEGFR and inhibits their kinase activities and also importantly G_M3_ ganglioside negatively regulates insulin receptor and causes partial insulin resistance [27]. Sulfatides (SM4s) are sulfoglycolipid that have specific functions in both the central nervous system and visceral organs [28] such as ligand binding to cell membrane [29] and their expressions are altered in many cancer types [30], [31]. Like other lysosomal storage diseases, Tay–Sachs display similar cellular pathological phenotypes that resulted from altered cellular processes such as reduction in autophagy, changes in calcium homeostasis, ER defects, mitochondrial dysfunctions and inhibited lipid trafficking [23]. Since specific gangliosides and sulfatides are accumulated in newly generated HexA^−/−^ Neu4^−/−^ Neu1^−/−^ and HexA^−/−^ Neu1^−/−^ mice, these models can be studied further to reveal how abovementioned cellular pathways are affected by neuraminidase-1 deficiency, b-series gangliosides and/or sulfatide alteration with or without neuraminidase-4.

## 5 Conclusion

With the analysis of HexA^−/−^ Neu4^−/−^ Neu1^−/−^ mice, we conclude that neuraminidase-1 contributes to the degradation of b-series gangliosides but not the metabolic bypass in HexA^−/−^ mice. Since, HexA^−/−^ Neu1^−/−^ and HexA^−/−^ Neu4^−/−^ Neu1^−/−^ mice have 10% of normal activity neuraminidase-1, we speculate that the level might be still high enough to degrade G_M2_ ganglioside in mice causing no accumulation. To reveal the exact effect of neuraminidase-1 on ganglioside degradation *in vivo*, a previously generated knockout mouse model of neuraminidase-1 can be used to obtain HexA^−/−^ Neu1^−/−^ mice [16]. Our study indicates that not only neuraminidase-4 and neuraminidase-1 but also other neuraminidases such as neuraminidase-2 and/or neuraminidase-3 may be involved in ganglioside degradation pathway in mouse. Therefore, new mouse models with combined deficiency of Hexosaminidase A and neuraminidase Neu2/Neu3 can be used to enlighten the roles of neuraminidase(s) in mice ganglioside degradation pathway which is different from human.
